# Simplified Surface Treatments for Ceramic Cementation: Use of Universal Adhesive and Self-Etching Ceramic Primer

**DOI:** 10.1155/2018/2598073

**Published:** 2018-12-31

**Authors:** Heloísa A. B. Guimarães, Paula C. Cardoso, Rafael A. Decurcio, Lúcio J. E. Monteiro, Letícia N. de Almeida, Wellington F. Martins, Ana Paula R. Magalhães

**Affiliations:** Restorative Dentistry, Brazilian Dental Association, Goiânia 74325-110, Brazil

## Abstract

The aim of this study was to evaluate the shear bond strength of resin cement and lithium disilicate ceramic after various surface treatments of the ceramic. Sixty blocks of ceramic (IPS e.max Press, Ivoclar Vivadent) were obtained. After cleaning, they were placed in polyvinyl chloride tubes with acrylic resin. The blocks were divided into six groups (n=10) depending on surface treatment: H/S/A - 10% Hydrofluoric Acid + Silane + Adhesive, H/S -10% Hydrofluoric Acid + Silane, H/S/UA - 10% Hydrofluoric Acid + Silane + Universal Adhesive, H/UA- 10% Hydrofluoric Acid + Universal Adhesive, MBEP/A - Monobond Etch & Prime + Adhesive, and MBEP - Monobond Etch & Prime. The light-cured resin cement (Variolink Esthetic LC, Ivoclar Vivadent) was inserted in a mold placed over the treated area of the ceramics and photocured with an LED for 20 s to produce cylinders (3 mm x 3 mm). The samples were subjected to a shear bond strength test in a universal test machine (Instron 5965) by 0.5 mm/min. ANOVA and Tukey tests showed a statistically significant difference between groups (p<0.05). The results of the shear strength test were H/S/A (9.61±2.50)^A^, H/S (10.22±3.28)^A^, H/S/UA (7.39±2.02)^ABC^, H/UA (4.28±1.32)^C^, MBEP/A (9.01±1.97)^AB^, and MBEP (6.18±2.75)^BC^. The H/S group showed cohesive failures, and the H/UA group was the only one that presented adhesive failures. The conventional treatment with hydrofluoric acid and silane showed the best bond strength. The use of a new ceramic primer associated with adhesive bonding obtained similar results to conventional surface treatment, being a satisfactory alternative to replace the use of hydrofluoric acid.

## 1. Introduction

Currently, several techniques and materials, such as composite resin and porcelain, have been used to correct aesthetic problems. The increasing popularity of the use of ceramic restorations for esthetic treatments is attributed to their superior optical properties, translucency, high mechanical properties, and improved esthetics [1]. Several ceramic systems are available, and glass ceramics reinforced by lithium disilicate have shown excellent clinical outcomes with great optical/mechanical properties and high survival rates over time [2].

The bond established between the ceramic material and the tooth structure is extremely important for success and longevity of ceramic restorations. To achieve a strong and durable bond, it is important to understand the ceramic's internal structure to select the best surface treatment, resin cement, and adhesive system [3]. For veneer cementation, the light-cured resin cement is preferable due to the number of colors available and long-term color stability [4].

For ceramic surface treatment, it is important to create a micromechanical interlock between the ceramic and the resin cement [5, 6]. Although the surface treatment with HF and silane is widely used and accepted for lithium disilicate ceramics [7–12], other alternatives have been proposed to enhance the bond strength between ceramic and resin cement.

The introduction of universal adhesives presents a new simplified approach for this procedure. They contain silane and a monomer called 10-methacryloyloxydecyl dihydrogen phosphate (MDP) that helps bond the ceramic to the resin cement chemically, simplifying the bonding procedure, providing the versatility of a single-bottle product, and reducing the procedure time [13]. Although recent studies [14, 15] have shown that the silane incorporated in a universal adhesive does not seem to produce the same adhesive strength as a silane agent applied separately, more studies are necessary to evaluate new strategies of cementation with these adhesives.

Even though it is highly used, the HF is a caustic and dangerous substance and presents a risk when contacting unprotected skin [16]. A self-etching ceramic primer (Monobond Etch & Prime, Ivoclar Vivadent) has been introduced as a single-component alternative to HF etching/silane routine surface treatment. The novel material aims to eliminate the risks associated with the HF acid as well as reduce the time required and the technique sensitivity of ceramics etching [17, 18]. Until now, few studies were available in the literature on the bonding efficiency of lithium disilicate ceramics to luting resin cements with this surface treatment. Some preliminary findings [18–20] showed that this self-etching ceramic primer presents a performance similar to that of the conventional surface treatment, but other authors showed that conventional treatment resulted in higher bond strengths than a self-etching ceramic primer [21, 22]. However, the use of this new ceramic primer should also be tested with various protocols.

Bonding effectiveness may directly influence the clinical success of ceramic restorations. It is important to identify the most reliable and effective surface treatment for ceramic before cementation. Therefore, the aim of this study was to investigate the influence of simplified ceramic surface treatments on shear bond strength of resin-luting cement and lithium disilicate ceramic. The null hypothesis tested was that various surface treatments and adhesive protocols will have no significant influence on the shear bond strength between resin cement and lithium disilicate ceramic.

## 2. Materials and Methods

The materials used and their respective compositions and batch numbers are displayed in Table 1.

### 2.1. Specimen Preparation

Sixty blocks of lithium disilicate-based ceramic (IPS e.max Press, Ivoclar Vivadent) were produced according to manufacturer instructions. The blocks were 8 mm tall, 8 mm wide, and 1 mm thick. To standardize the ceramic blocks, a wax pattern (VKS Gray Wax, Yeti Dental Produkte, Engen, Germany) was made in the dimensions of future blocks for ceramic injection. Dimensions were checked with a digital caliper (Mitutoyo Corporation, Tokyo, Japan).

Ceramic specimens were sandblasted with 50 micrometers of aluminum oxide particles for 15 s, then cleaned in an ultrasonic bath, and immersed first in distilled water and then in 92.8% ethanol, for 10 minutes each. Then they were placed in polyvinyl chloride (PVC) tubes (15 mm thick and 20 mm in diameter) with acrylic resin (Jet, Lapa, Rio de Janeiro, Brazil).

### 2.2. Ceramic Surface Treatments

The luting protocols for the ceramic surface treatment were performed according to the groups to which the specimens belonged, described in Table 2. Sixty specimens were divided into 6 groups (n=10).

In the first group (H/S/A), the ceramic surface was etched for 20 s with 10% HF (Condac, FGM), washed with an air/water spray for 30 s, and then dried with an air spray. The silane (Monobond N, Ivoclar Vivadent) was applied with a microbrush and allowed to react for 60 s. Subsequently, the excess was dispersed with a strong stream of air to ensure the solvent's evaporation. Finally, the adhesive agent (AdheSE Bonding Agent, Ivoclar Vivadent) was applied with a microbrush in a thin layer and polymerized using an LED curing unit (Bluephase, Ivoclar Vivadent) for 20 s. For the H/S group, the same protocol was followed; however, no adhesive was applied, just HF and silane. For the third group (H/S/UA), after etching with HF and silane application, a universal adhesive (SingleBond Universal, 3M ESPE) was applied in a thin layer with a microbrush and polymerized using an LED light-curing unit (Bluephase, Ivoclar Vivadent). For the MBEP/A group, a new ceramic primer (Monobond Etch & Prime, Ivoclar Vivadent) was applied without the use of HF or silane. Initially, the primer was applied with a microbrush with friction for 20 s and then it was allowed to sit for 40 s, and the surface was washed abundantly with an air/water spray followed by drying with an air spray for 10 s. Afterward, the adhesive agent (AdheSE Bonding Agent, Ivoclar Vivadent) was applied with a microbrush in a thin layer and polymerized using an LED light-curing unit (Bluephase, Ivoclar Vivadent) for 20 s. For the MBEP group, only the ceramic primer was applied (Monobond Etch & Prime, Ivoclar Vivadent) according to the method for the last group; however, no adhesive was applied.

### 2.3. Resin Cement Cylinders Production

A special metal device was used to fix a Teflon mold, with a cylindrical cavity 3 mm wide and 3 mm deep, to the pretreated ceramic surface. The light-cured resin cement (Variolink Esthetic LC, Ivoclar Vivadent), color Neutral (translucent), was injected into the mold. The excess cement was removed using a microbrush, and the luting resin cement was photocured using an LED curing unit (Bluephase, Ivoclar Vivadent) operating at 1200 mW/cm^2^ in high-power mode for 20 s. The mold was disassembled and resultant rods were examined for any composite flashes, which were removed with a sharp blade.

### 2.4. Shear Bond Strength Test

The samples were stored in distilled water at 37°C for 24 h. In this study, the same device was used as in a previous study [23], with a metal strip around the cement cylinder to minimize the flexural stresses. The machine's semicircular metal attachment applied shear forces at the cement-ceramic interface, running at a crosshead speed of 0.5 mm/min, until complete failure. The maximum load to failure (in Newtons) was recorded, and the shear bond strength (in MPa) was calculated by dividing the failure load by the bonding area (mm^2^), which was calculated by measuring the cement cylinder's diameter at two points with a digital caliper. The same operator carried out all procedures to avoid interoperator variability. All manufactured specimens were tested for shear bond strength, as no pretest failures were observed.

The debonded specimens were examined under an optic microscope (Discovery V8 Stereo, Carl Zeiss Microimaging GmbH, Jena, Germany) to determine the failure mode. They were classified as adhesive failure, between resin cement and ceramic (A), mixed failure (M), and cohesive in resin cement (CR) or cohesive in ceramic (CC).

### 2.5. Scanning Electron Microscope (SEM)

SEM images of the lithium disilicate-based ceramic (IPS e.max Press) surface were captured at various magnifications to evaluate the etching pattern/micromorphology produced by each treatment (no treatment, HF 10% or Monobond Etch & Prime) used according to manufacturers' instructions.

### 2.6. Statistical Analysis

Data obtained on shear bond strength was analyzed in Stat Plus (Mac v.6.2.21 (Analysoft, Inc, Atlanta, USA). Initially, the data were analyzed for homogeneity (Levene's test) and normality (Kolmogorov-Smirnov test). Due to its parametric and homogeneous distribution, the ANOVA test was used with multiple comparisons with the post hoc Tukey test (*α* = 0.05).

## 3. Results

The mean and standard deviation values of each group's shear bond strength are summarized in Table 3. Significant statistical differences were observed in shear bond strength for the surface treatments (p<0.05). The surface treatment with hydrofluoric acid and silane (H/S group) showed the highest values of shear bond strength; however, it did not differ statistically from H/S/A, MBEP/A and H/S/UA. The use of only Monobond Etch & Prime (MBEP group) as a surface treatment led to significantly lower values of bond strength than in the MBEP/A group and was statistically similar to the H/UA group, which obtained the lowest values of bond strength. The use of silane prior to application of the universal adhesive (H/S/UA group) promoted higher values than in the group in which the universal adhesive was used without silane (H/UA).

Failure mode was also influenced by surface treatment, according to Table 4. Cohesive failures in ceramic were not observed. The H/S/A, H/S/UA, and MBEP/A groups showed the most mixed failures and a small number of cohesive failures in resin cement. These mixed failures usually presented resin cement in the border areas of the specimen and debonding in the center of the specimen. The H/S group only showed cohesive failures in resin cement, and H/UA was the only group that showed adhesive failures.

**Figure 1:**
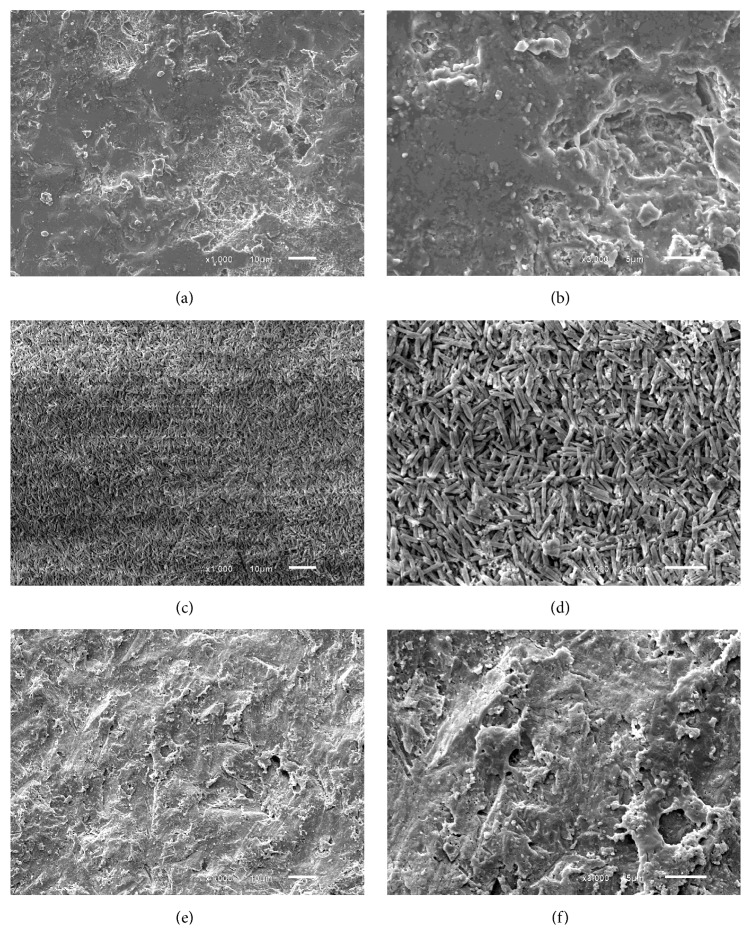
SEM images of nonetched and etched IPS e.max ceramic surfaces after different conditioning. (a) x1000 magnification, ceramic surface before etching. (b) x3000 magnification, ceramic surface before etching. (c) x1000 magnification, etching with HF 10% for 20 seconds. (d) x3000 magnification, etching with HF 10% for 20 seconds. (e) x1000 magnification, etching with Monobond Etch & Prime according to the manufacture. (f) x3000 magnification, etching with Monobond Etch & Prime according to the manufacture.

SEM analysis (Figure 1) showed the difference between the ceramic with no treatment and the etching pattern produced by HF and by Monobond Etch & Prime surface treatment. After surface treatment with HF, it is possible to observe a deeper etching pattern with glassy dissolution and exposition of crystals. When the self-etching primer was used, e etching pattern was more superficial, showing less micromechanical retention with smaller glassy dissolution and without crystal exposition.

## 4. Discussion

The clinical success of a ceramic restoration depends on the quality and durability of the bond between ceramic and resin cement [24]. The protocol established for lithium disilicate-based ceramic cementation is the etching with HF and the application of a silane agent [25]. In the present study, various surface treatments were used, simplified or not, and the results showed that multiple surface treatments and adhesive protocols promoted significant changes in shear bond strength, disproving the null hypothesis.

In the cementation of lithium disilicate-based ceramics, the surface treatment with HF is extremely important to promote irregularities and create a surface with micropores by partially dissolving the glass phase, leaving behind an active surface rich in silica [3, 7]. The silane coupling agent establishes adhesion between the inorganic phase of the ceramic and the organic phase of the resin cement, forming a siloxane bond [11, 26]. The use of silane after etching with HF is indispensable; however, the use of the adhesive is still controversial. The groups that had application of the silane (H/S/A, H/S, and H/S/UA) did not present significant statistical differences among themselves; therefore, the use of the adhesive appears dispensable; this finding corroborates with those of Garboza et al. [27]. The use of HF and silane seems to be the ideal protocol because it requires fewer steps and reduces the risk of failure.

When the failure modes (Table 4) were observed, only the H/S group showed only cohesive failures in resin cement. According to Chen et al. [28], this may suggest that the adhesive interface was very strong, and the application of adhesive after silane probably weakened the interface. On the other hand, Scherrer, Cesar, and Swain [29] affirm that, when cohesive and mixed cohesive/adhesive failures occur, the bond strength obtained is not representative of the interface adhesion but reflects the strength of the materials being tested. As only one resin cement was evaluated, the differences in bond strength obtained may represent the different surface treatments performed. According to DeHoff, Anusavice, and Wang [30], due to the known variation in bond strength with specimen preparation and design, data on the same systems may show great variability in mean and large standard deviations. Thus, these tests should be used, as in this study, as a tool to compare materials or surface treatments to determine the effect of changing some variable for the same system and not to determine the real bond strength between resin cement and ceramic [30].

The H/S/UA and H/UA groups were statistically similar; however, when only universal adhesive was used after HF, shear bond strength values decreased. Although the universal adhesive used contains silane and 10-methacryloxydecyl dihydrogen phosphate (10-MDP), the additional salinization step enhances chemical bonding to the exposed hydroxyl groups and surface wettability with resin impregnation, which has been shown in other studies, even in the long term [31–33]. Moreover, only the H/UA group exhibited adhesive failures, which corroborates the fact that only the universal adhesive after HF is ineffective in preparing the ceramic surface because the adhesive interface proved to be flawed and fragile. When HF, silane, and adhesive were used, the adhesive bonding (H/S/A) was more effective than the universal adhesive (H/S/UA). This finding is in accordance with those of Garboza et al. [27] and can be explained by the fact that the hydrophilic part of the universal adhesive might negatively affect the bond strength. Moreover, the silane contained in universal adhesive may have increased the hydrophilicity, thereby predisposing the adhesive layer to hydrolytic degradation.

The self-etching ceramic primer (Monobond Etch & Prime) contains ammonium polyfluoride and silane in a single step. This new material aims to eliminate the toxic potential of HF and minimize the technical sensitivity of the cementation process [27]. However, the ammonium polyfluoride promotes a weaker etching pattern in the ceramic surface than HF [9, 27]. Previous studies [27, 34] showed that this primer was efficient in conditioning vitreous ceramics, presenting bond strength comparable to that of the conventional treatment. However, the conventional treatment still showed superior results, remaining a gold standard for ceramic surface treatment. A recent study [22] showed that HF/silane resulted in higher mean microshear bond strength than Monobond Etch & Prime for lithium disilicate and feldspathic ceramics; however, Monobond Etch & Prime had a more stable bond after aging.

In the present study, the MBEP group was statistically inferior to the conventional treatment (H/S), corroborating with previous studies that included shear bond evaluation [17, 21, 34]. This result is probably due to the etching pattern promoted by HF. In SEM images (Figures 1(c) and 1(d)), after surface treatment of HF 10%, it was possible to observe a porous surface with exposure of lithium disilicate crystals on the ceramic surface, resulting in more surface area for resin bonding and promoting better chemical bonding via a silane coupling agent [35]. In Figures 1(e) and 1(f), it is possible to observe that MBEP showed almost no etching depth power, probably because of its weaker etching agent (ammonium polyfluoride), resulting in less micromechanical retention of resin cement. This finding corroborates with those of Lopes et al. [21], who evaluated the etching pattern of lithium disilicate ceramics under a field emission scanning electron microscope and showed that use of Monobond Etch&Prime resulted in the least pronounced etching pattern.

Despite these findings, when a conventional adhesive was used after ceramic primer (MBEP/A group), the resulting bond strength was similar to that of conventional surface treatment (H/S). Although the mechanism of action and adhesion of this self-etching ceramic primer is not very clear, the use of bonding adhesive promotes better interaction between ceramic and resin cement, presenting even more mixed failures than the MBEP group, probably due to the better chemical bond established. The unfilled adhesive probably promotes the formation of a more compatible and stronger interaction between the pretreated ceramic and the resin cement. Resin adhesives are usually made up of hydrophobic dimethacrylates, which may covalently bond to silane and cement materials by means of ester bonds. Consequently, a strong intermolecular chemical interaction between ceramic and cement could be achieved, leading to the formation of a homogeneous tertiary monoblock [36, 37]. These results are still favorable because, even though the application of the adhesive agent is required, the use of the primer eliminates the use of HF and contributes to a safer procedure.

A restoration in the oral cavity is challenged in many ways: it is subjected to complex occlusal forces, immersed in saliva and exposed to food and beverages with various pH, chemistries, and temperatures. Numerous laboratory tests have attempted to simulate oral conditions in order to predict clinical bonding performance. However, no single laboratory test is able to adequately predict the clinical performance of resin-ceramic bonding [5].

The most common tests to evaluate resin-ceramic bonding measurements are shear and tensile bond strength tests [5]. As in other studies [10, 19, 26] the shear bond strength test was used in this work, even though it is known that “macro” bonding tests, due to the bigger adhesion area, tend to result in lower bond strength values [38]. This method is commonly used for ceramics bond strength evaluation, not only because it is a quick and repeatable testing option but also because it is difficult to section the ceramic for microtensile testing [5]. The use of the stainless steel tape, instead of a knife edge or a looped orthodontic wire system, for the test is justified by the possibility of reducing the stress-concentration magnitude adjacent to the interface, and the tensile and compressive stresses produced in the interface are smaller than those obtained from the other systems [23, 30, 39].

One of this study's limitations was the use of one type of light-cured resin-luting cement. Tests with multiple types of resin cements, including self-adhesive and dual-cured cements, can be interesting and could be a point for further research. Moreover, studies with long-term water storage and thermocycling are necessary to evaluate mainly the new materials. Finally, clinical studies are needed to evaluate this material's clinical performance. The use of a new self-etch ceramic primer associated with adhesive bonding is an effective alternative to simplify the clinical procedures presenting a performance similar to that of conventional surface treatment for lithium disilicate ceramics.

## 5. Conclusions

Even with the present study's limitations, it is possible to conclude that the surface treatment with HF and silane is an effective and simple alternative to luting lithium disilicate ceramics; the use of universal adhesive did not exempt the application of a silane, and the new ceramic self-etching primer is an effective alternative for simplified ceramic surface treatment when an adhesive agent is applied after it.

## Figures and Tables

**Table 1:** Materials used in this study and respective manufactures, compositions, and batch numbers.

**Material**	**Manufacture**	**Composition**	**#Batch number**
Condac	FGM, Joinville, Brazil	10% hydrofluoric acid	060917
Monobond N	Ivoclar Vivadent, Shaan, Liechtenstein	Ethanol, 3-trimethoxysilylpropyl methacrylate, 10-MDP, disulfide acrylate	V43819
AdheSE Bonding Agent	Ivoclar Vivadent, Shaan, Liechtenstein	Dimethacrylates, Hydroxyethyl methacrylate, Highly dispersed silicon dioxide, Initiators and stabilizers	U54846
Single Bond Universal	3M ESPE, Saint Paul, USA	Organophosphate monomer (MDP), Bis-GMA, HEMA, Vitrebond copolymer, ethanol, water, initiators, silane	507329
Monobond Etch & Prime, self etching glass ceramic primer	Ivoclar Vivadent, Shaan, Liechtenstein	Tetrabutyl ammonium dihydrogen trifluoride, methacrylated phosphoric acid ester, trimethoxysilylpropyl methacrylate, alcohol, water	V09353
Variolink Esthetic LC	Ivoclar Vivadent, Shaan, Liechtenstein	Bis-GMA, UDMA, TEGDMA, ytterbium trifluoride, boroaluminofluorosilicate glass, spheroidal mixed oxide, benzoylperoxide, stabilizers, pigments	V37749

**Table 2:** Group codes and surface treatments of ceramic.

**Groups**	**Surface treatment**
**H/S/A **	10% Hydrofluoric Acid + Silane + Adhesive
**H/S**	10% Hydrofluoric Acid + Silane
**H/S/UA**	10% Hydrofluoric Acid + Silane + Universal Adhesive
**H/UA**	10% Hydrofluoric Acid + Universal Adhesive
**MBEP/A**	Monobond Etch & Prime + Adhesive
**MBEP**	Monobond Etch & Prime

**Table 3:** Means (MPa), standard deviations, and confidence intervals of shear bond strength for each group.

**Groups**	**Shear bond strength (mean)**	**Standard deviation**	**Confidence interval**
**H/S/A**	9.60^A^	2.50	7.81-11.39

**H/S**	10.22^A^	3.28	7.89-12.57

**H/S/UA**	7.39^A,B,C^	2.02	5.98-8.84

**H/UA**	4.28^C^	1.32	3.33-5.23

**MBEP/A**	9.00^A,B^	1.97	7.59-10.41

**MBEP**	6.18^B,C^	2.75	4.21-8.15

Values followed by different letters present statistical difference (p<0.05).

**Table 4:** Distribution of failure modes in percentage (%) and absolute numbers (n) after shear bond strength test for all tested groups.

**Groups**	**Adhesive Failure - **%** (n)**	**Cohesive Failure - **%** (n)**	**Mixed Failure - **%** (n)**	**Pre-test failures **%** (n)**
**H/S/A**	0 (0)	30 (3)	70 (7)	0 (0)
**H/S**	0 (0)	100 (10)	0 (0)	0 (0)
**H/S/UA**	0 (0)	20 (2)	80 (8)	0 (0)
**H/UA**	20 (2)	0 (0)	80 (8)	0 (0)
**MBEP/A**	0 (0)	20 (2)	80 (8)	0 (0)
**MBEP**	0 (0)	90 (9)	10 (1)	0 (0)

## Data Availability

The data used to support the findings of this study are included within the article and necessary explanations in relation to this can be asked with the corresponding author.
